# Immunogenic cell death induced by a new photodynamic therapy based on photosens and photodithazine

**DOI:** 10.1186/s40425-019-0826-3

**Published:** 2019-12-16

**Authors:** Victoria D. Turubanova, Irina V. Balalaeva, Tatiana A. Mishchenko, Elena Catanzaro, Razan Alzeibak, Nina N. Peskova, Iuliia Efimova, Claus Bachert, Elena V. Mitroshina, Olga Krysko, Maria V. Vedunova, Dmitri V. Krysko

**Affiliations:** 1 Cell Death Investigation and Therapy Laboratory, Department of Human Structure and Repair, Ghent University, Ghent, Belgium; 2 Institute of Biology and Biomedicine, National Research Lobachevsky State University of Nizhni Novgorod, Nizhni Novgorod, Russian Federation; 3 Department for Life Quality Studies, Alma Mater Studiorum-University of Bologna, Rimini, Italy; 4 Cancer Research Institute Ghent, Ghent, Belgium; 5 Upper Airways Research Laboratory, Department of Head and Skin, Ghent University, Ghent, Belgium

**Keywords:** Immunogenic cell death, ICD, Cancer, Photodynamic therapy, Ferroptosis, Ferrostatin-1, Necroptosis, Apoptosis

## Abstract

**Background:**

Anti-cancer therapy is more successful when it can also induce an immunogenic form of cancer cell death (ICD). Therefore, when developing new treatment strategies, it is extremely important to choose methods that induce ICD and thereby activate anti-tumor immune response leading to the most effective destruction of tumor cells. The aim of this work was to analyze whether the clinically widely used photosensitizers, photosens (PS) and photodithazine (PD), can induce ICD when used in photodynamic therapy (PDT).

**Methods:**

Cell death in murine glioma GL261 or fibrosarcoma MCA205 cells was induced by PS- or PD-PDT and cell death was analyzed by MTT or flow cytometry. Intracellular distribution of PS and PD was studied by using the laser scanning microscope. Calreticulin exposure and HMGB1 and ATP release were detected by flow cytometry, ELISA and luminescence assay, respectively. Immunogenicity in vitro was analyzed by co-culturing of dying cancer cells with bone-marrow derived dendritic cells (BMDCs) and rate of phagocytosis and maturation (CD11c^+^CD86^+^, CD11c^+^CD40^+^) of BMDCs and production of IL-6 in the supernatant were measured. In vivo immunogenicity was analyzed in mouse tumor prophylactic vaccination model.

**Results:**

We determined the optimal concentrations of the photosensitizers and found that at a light dose of 20 J/cm^2^ (λex 615–635 nm) both PS and PD efficiently induced cell death in glioma GL261 and fibrosarcoma MCA205 cells. We demonstrate that PS localized predominantly in the lysosomes and that the cell death induced by PS-PDT was inhibited by zVAD-fmk (apoptosis inhibitor) and by ferrostatin-1 and DFO (ferroptosis inhibitors), but not by the necroptosis inhibitor necrostatin-1 s. By contrast, PD accumulated in the endoplasmic reticulum and Golgi apparatus, and the cell death induced by PD-PDT was inhibited only by z-VAD-fmk. Dying cancer cells induced by PS-PDT or PD-PDT emit calreticulin, HMGB1 and ATP and they were efficiently engulfed by BMDCs, which then matured, became activated and produced IL-6. Using dying cancer cells induced by PS-PDT or PD-PDT, we demonstrate the efficient vaccination potential of ICD in vivo.

**Conclusions:**

Altogether, these results identify PS and PD as novel ICD inducers that could be effectively combined with PDT in cancer therapy.

## Introduction

Over the last decade, it has become clear that anti-cancer therapy is more successful when it can also induce an immunogenic form of cell death (ICD). The ICD concept suggests that activation of an immune response specific for cancer cells generates a strong and long-lasting anti-cancer immunity [1, 2]. ICD is characterized by the emission of immuno-stimulatory molecules, including damage-associated molecular patterns (DAMPs) such as exposure of calreticulin (CRT) at the cell surface and release of HMGB1 and ATP [3–8]. These molecules function as adjuvants and contribute to the activation of antigen presenting cells (e.g. dendritic cells), which engulf dying cancer cells [2], leading to cross-presentation of antigenic peptides to CD8^+^ T cells, one of the main driving forces of anti-tumor immune responses [9, 10].

Recently, efforts have focused on the validation of approved conventional anticancer therapies (e.g. anthracyclines, γ-irradiation) to induce ICD in cancer cells [2, 11, 12]. Photodynamic therapy (PDT), a clinically approved and minimally invasive therapeutic procedure [13], has been added to a list of strategies that can induce ICD in cancer cells [7, 14, 15]. PDT involves the administration of a photosensitizer followed by irradiation at a wavelength corresponding to an absorbance band of the photosensitizers. Light activation of the photosensitizer transfers energy to molecular oxygen to produce singlet oxygen, a highly reactive toxic species that rapidly reacts with cellular components, causing oxidative damage and ultimately leading to the death of tumor cells. This procedure is often associated with secondary effects such as damage to the microvasculature and induction of a local inflammatory reaction [16]. An ideal photosensitizer should accumulate selectively in tumors, have low dark toxicity and be easy to synthesize [17–19]. Importantly, it should also be able to induce ICD in cancer cells. Only a few photosensitizers are known to induce ICD, namely hypericin [7, 20], 5-ALA [21], Rose Bengal acetate [14] and glycoconjugated chlorin [15]. The goal of the present study was to investigate whether clinically approved photosens [PS, phthalocyanines complexed with aluminum [22–24];] and photodithazine [PD, [25, 26]] (Additional file 1: Figure S1A and S1B) can induce ICD in vitro and in vivo in a tumor prophylactic vaccination model.

In this study we first characterized the cell death type induced by PS-PDT and PD-PDT and the cellular distribution of PS and PD. We demonstrate that glioma GL261 and fibrosarcoma MCA205 cells induced to die by PS-PDT or PD-PDT emit DAMPs such as CRT exposure at the plasma membrane and release of ATP and HMGB1. These dying cancer cells are efficiently phagocytosed by BMDCs, inducing their maturation and activation in a manner that depends on the ratio of the two cell types, as evidenced by increased surface expression of CD40 and CD86. Using cancerous cells after induction of cell death by PS-PDT or PD-PDT, we demonstrate the effective vaccination potential of ICD in a mouse tumor prophylactic vaccination model.

## Materials and methods

### Cell lines

Murine glioma GL261 and fibrosarcoma MCA205 cells were cultured at 37 °C under 5% CO_2_ in DMEM and RPMI, respectively, containing 4.5 g/L glucose and supplemented with 2 mM glutamine, 100 μM sodium pyruvate, 100 units/ml penicillin, 100 μg/L streptomycin and 10% fetal bovine serum (FBS, Fisher Scientific, 10,082,147).

### Spectra acquisition

The following photosensitizers were used: photosens (PS, a mixture of di-, tri- and tetrasubstituted fractions of aluminum phthalocyanine, the number of sulfo groups is 3.4; NIOPIK, Russia) and photodithazine (PD, bis-N-methylglucamine salt of chlorin *e*6; Veta-grand, Russia). Spectra of absorption and fluorescence emission of PS and PD were registered using Synergy MX Microplate Reader (BioTek, USA) in black 96-well microplates with a clear glass bottom (Falcon Imaging; Corning, USA). Photosensitizer solutions were prepared in distilled water at 10 μg/ml. Absorption spectra were obtained in the range of 320–850 nm for PS and from 300 to 700 for PD. Fluorescence was excited at 405 nm and recorded in the range of 655–850 nm for PS and 600–850 nm for PD.

### PDT-induced cell death

Cell death was induced by PS- or PD-based PDT. For this, GL261 and MCA205 cells were first incubated with 1.4 μM PS or 1.2 μM PD and 1.5 μM PS or 1.8 μM PD, respectively, in serum-free medium for 4 h. Then the cells were irradiated with a light dose of 20 J/cm^2^ using a LED light source (λex 615–635 nm) in photosensitizer-free media. Cells loaded with photosensitizers were handled either in the dark or subdued light. After PDT, the cells were cultured in complete medium for the indicated period of time and cell death was analyzed by MTT or flow cytometry. Control cells were cultured in the same conditions but without photosensitizers or PDT.

The following blockers were used to inhibit cell death: the pan-caspase inhibitor carbobenzoxy-valyl-alanyl-aspartyl-[O-methyl]-fluoromethylketone (zVAD-fmk, 25 μM, Sigma-Aldrich), RIPK1 inhibitor necrostatin-1 s (Nec-1 s, 20 μM, Abcam), the inhibitor of ROS and lipid peroxidation ferrostatin-1 (Fer-1, 1 μM, Sigma-Aldrich) and the iron chelator, deferoxamine (DFO, 10 μM, Sigma-Aldrich). The cell death inhibitors were added together with the corresponding photosensitizer or DMSO and the cells were incubated for 4 h in serum-free conditions. Before PDT, the medium was replaced with complete medium containing the respective cell death inhibitor, the cells were irradiated with light at 20 J/cm^2^, and then they were incubated for 13 h.

### Cell death assay by flow cytometry and MTT

The cells were washed in Annexin V binding buffer and stained with SYTOX Blue Nucleic Acid Stain (Molecular Probes) and FITC Annexin V (Invitrogen). The assay was run on a BD FACSCanto flow cytometer. The data were analyzed using FlowJo software. MTT assay (AlfaAesar) was performed according to the manufacturer’s instructions and the optical density was measured at 570 nm.

### Accumulation dynamics and subcellular distribution of PS and PD

Intracellular distribution of PS and PD was studied by using the LSM 710 Axio Obzerver Z1 DUO NLO laser scanning microscope (Carl Zeiss, Germany). The images were obtained using a LD C-Apochromat water immersion objective lens 40×/1.1. The GL261 cells were seeded in 96-well glass-bottom plates (Corning, USA) at 10^4^ cells per well and grown overnight. The cells were then incubated with 10 μM photosensitizers in serum-free culture medium for 1–4 h, followed by washing with PBS and confocal image acquisition. The fluorescence of PS and PD was excited at 633 nm and recorded in the range of 650–735 nm.

For colocalization analysis of PS and PD after 3.5 h of incubation of GL261 cells with the respective photosensitizer, the following dyes were added for 30 min (ThermoFisherScientific): 0.5 μM LysoTracker Green DND-26 for lysosomes, 0.5 μM ER-Tracker for endoplasmic reiculum, 0.5 μM MitoTracker Green FM for mitochondria, 5 μM BODIPY FL C5-ceramide complexed to BSA for Golgi apparatus. Dyes were added to living cells that had been incubated with the photosensitizers. Staining was performed according to the manufacturer’s instructions. Fluorescence of stained organelles was excited by an argon laser at 488 nm and registered in the range of 500–560 nm.

### Flow cytometry analysis of CRT exposure at the cell surface

GL261 and MCA205 cells were stimulated by either PS-PDT or PD-PDT as described above. After 1.5 h and 3 h of incubation, the cells were collected and then washed with ice-cold FACS buffer (PBS, BSA 1%, FBS 2%). After centrifugation (1500 rpm 4 °C 5 min), they were resuspended in ice-cold FACS buffer with anti-calreticulin antibody (ab210431; 0.5 mg/ml) or isotype control rabbit IgG (Ab208150; 0,5 mg/ml). The cells were incubated for 40 min at 4 °C and then resuspended in 200 μL of ice-cold FACS buffer and stained with 0.8 μM Sytox Green (Molecular Probes, S7020). Finally, the samples were analyzed by flow cytometry on a BD FACS Canto II. Analysis was performed using FlowJo software (v.10.0.8). The surface exposure of CRT was determined in Sytox Green negative cells.

### HMGB1 release

After the indicated time points, supernatant was collected and cleared from dying tumor cells by centrifugation, frozen at − 20 °C for later HMGB1 quantification by an ELISA kit (IBL-Hamburg). All assays were performed in accordance with the respective manufacturers’ instructions and HMGB1 was quantified using Tecan Spark® 20 M microplate multimode reader. The data were analyzed with a Four Parameter Logistic Curve Fit.

### ATP release

GL261 and MCA205 cells were treated with PS-PDT or PD-PDT as described above and incubated for 24 h in a medium with 2% FBS. Then supernatants were collected and centrifuged at 15,000 rpm at 4 °C for 3 min. The supernatants were either stored at − 80 °C or used immediately for ATP measurements. ATP analysis was done by using CellTiter-Glo® Luminescent Cell Viability Assay kit (Promega, G7571) as described by the manufacturer. The luminescence was measured on a Tecan Spark® 20 M microplate multimode reader.

### Generation of mouse bone-marrow derived dendritic cells

Over 10 days, bone-marrow derived dendritic cells (BMDCs) were differentiated from the femurs and tibias of C57BL/6 J mice at the age of 7–9 weeks using RPMI medium (GIBCO) supplemented with 5% heat-inactivated fetal calf serum, 20 ng/ml mGM-CSF, 1% L-glutamine and 50 μM 2-mercapthoethanol, 1 mM pyruvate. Fresh culture medium was added on day 3, and on day 6 and 9 the medium was refreshed.

### Phagocytosis assay

Target GL261 and MCA205 cells were labeled with 1 μM CellTracker Green CMFDA (Molecular Probes) in serum-free media for 30 min and then either left untreated or induced to die by PS-PDT or PD-PDT, as described above. The cells were collected, washed, and co-cultured with BMDCs in ratios of 1:1, 1:5 or 1:10 for 2 h. Next, the co-cultured cells were harvested, incubated with a mouse Fc block (ThermoFisherScientific), immunostained with PE-Cy-anti-CD11c (BD PharMingen, 561,022), and finally analyzed by flow cytometry on a BD FACSCanto. Analysis was performed using FlowJo software (v.10.0.8). True uptake of CMFDA-labeled dead cell material by BMDCs was determined using a gating strategy that allows analysis of only single cells and was determined as CD11c CMFDA double-positive cells.

### Analysis of BMDCs maturation and IL-6 production

Immature murine BMDCs were isolated and cultured as described previously. Then BMDCs were co-incubated with dying GL261 or MCA205 cells stimulated with PS-PDT or PD-PDT as described above in ratios 1:1, 1:5 or 1:10 for 18 h. As a positive control, BMDCs were stimulated in parallel with 100 ng/ml of *E. coli* lipopolysaccharide (LPS). After co-culture for 18 h, the cells were collected, spun down (400×g, 6 min, 4 °C), and washed once in phosphate buffered saline (PBS, Life Technologies). Dead cells were excluded from the flow cytometry analysis by staining with SYTOX Blue (Molecular Probes, S11348). Maturation of BMDCs was analyzed by immunostaining with anti-CD11c PE-Cy7 (BD PharMingen), anti-CD86-eFluor 450 or -APC (eBioscience), anti-CD40 Pacific Blue (Biolegend), eFluor 45–anti-CD80-eFluor 450 (Thermo Fisher Scientific) and mouse Fc block (Thermo Fisher Scientific). After co-culturing BMDCs with the MCA205 cancer cells, the supernatants were collected and IL-6 was measured by ELISA (BioLegend).

### In vivo prophylactic tumor vaccination

Female C57BL/6 J mice (7–8 weeks old) were housed in specific pathogen-free conditions. All experiments were performed in accordance with the guidelines of the local Ethics Committee of Ghent University (ECD19/35).

Cell death in MCA205 cells was induced in vitro by PS-PDT, PD-PDT as described above. Next, the cells were collected, washed once in PBS, and re-suspended at the desired cell density in PBS. Mice were inoculated subcutaneously with 5 × 10^5^ dying MCA205 cells or with PBS on the left flank. On day 8 after vaccination, the mice were challenged subcutaneously on the opposite flank with 1 × 10^5^ live MCA205 cells. Tumor growth at the challenge site was monitored using a caliper for up to 4 weeks after the challenge. Mice were sacrificed when the tumors became necrotic or exceeded 2 cm^3^.

### Statistical analysis

Statistical analysis was performed in GraphPad Prism (v.6.0). Cell death was analyzed by ANOVA followed by t-criteria with Bonferroni correction. The phagocytosis assay was analyzed by two-way ANOVA. The results of the BMDC activation and maturation assay were analyzed by Mann-Whitney non-parametric t-test. Kaplan-Meier survival curves showing the timeline for tumor development were analyzed by log-rank Mantel-Cox test. Differences between tumor volumes on the mice in the vaccination experiments were analyzed by a non-parametric Mann-Whitney test.

## Results

### Spectral characteristics, cellular uptake and localization of PS and PD in cancer cells

First, we analyzed the absorption and fluorescence spectra of PD belonging to the chlorins derivatives. For PS, we observed the typical absorption and fluorescence spectra (Additional file 1: Figure S1A), which is in agreement with the previously published data [19]. On the other hand, for PD, absorption peaks were present in the short-wave (Soret band) and long-wave (Q-band) regions of the spectrum (Additional file 1: Figure S1B). Although PS and PD accumulated in GL261 glioma cells during in vitro incubation, their uptake rates and intracellular localizations differed significantly. PS had a lower rate of accumulation in GL261 cells than PD because it is a hydrophilic compound that enters cells by active endocytosis (Additional file 1: Figure S1C, S1D). Notably, incubation for 4 h was enough for both photosensitizers to accumulate to a significant extent in GL261 cells. Therefore, this incubation time was chosen for analysis of their photodynamic activities.

**Fig. 1:**
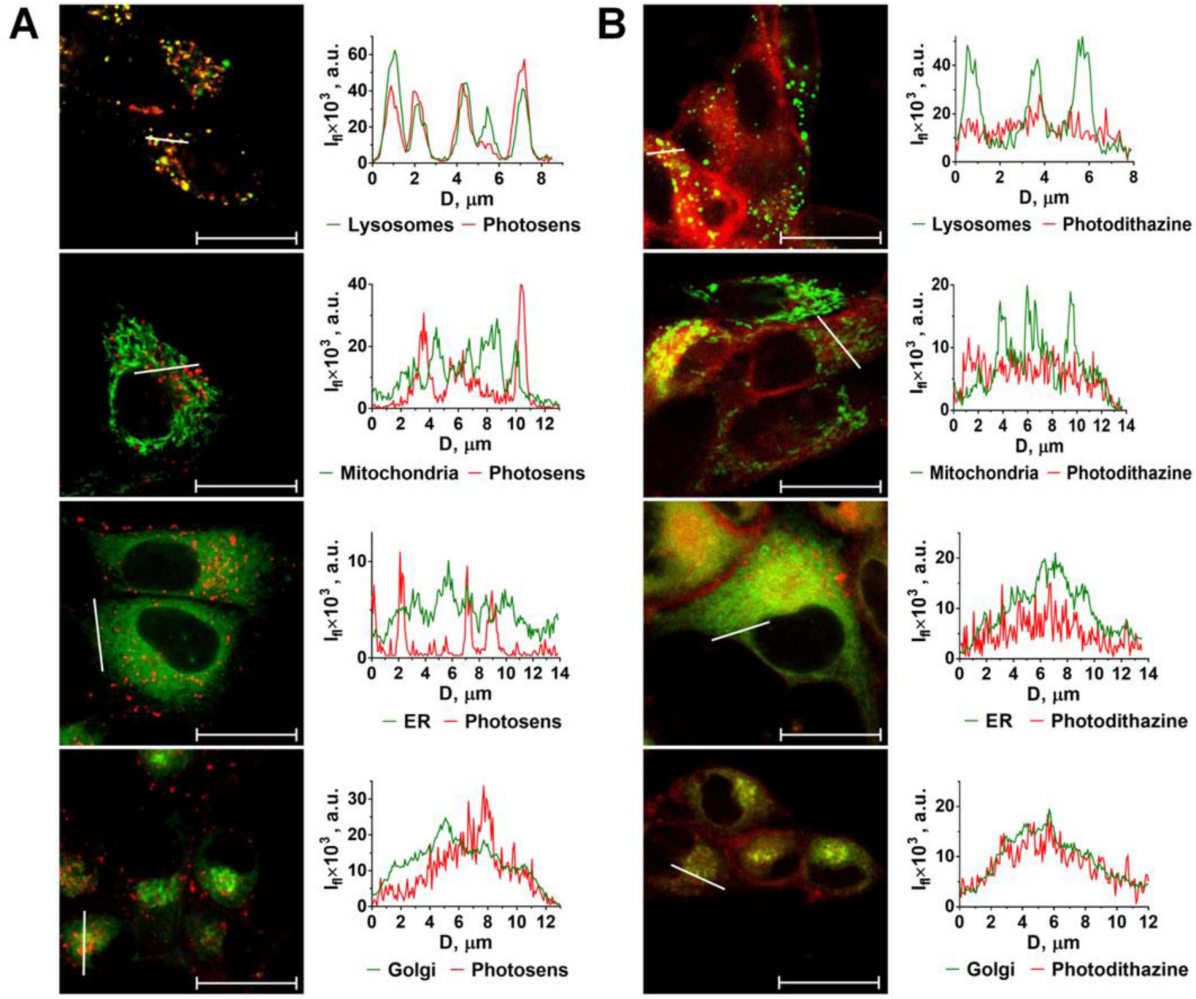
Subcellular distribution of photosens (PS) and photodithazine (PD) in cancer cells. The subcellular localization of PS (**a**) and PD (**b**) differ significantly as studied by confocal microscopy after 4 h of incubation (both at 10 μM) with GL261 cells. PS is mostly co-localized with lysosomes and, potentially, other intercellular vesicles (**a**). PS was not detected in mitochondria, ER, Golgi apparatus and nuclei. In contrast, PD accumulated mostly in the ER and Golgi apparatus (**b**). Fluorescence signal profiles along the lines indicated by the white arrow on the images with superimposed fluorescence channels. I_fl_: fluorescence intensity; D: distance along the specified segment. The following dyes were used: LysoTracker Green for lysosomes; MitoTracker Green for mitochondria; ER-Tracker for ER; BODIPY FL С5-ceramide for Golgi apparatus. Scale bars, 20 μm

It is known that the capacity to induce ICD is associated with localization of the photosensitizers or drugs in the ER and their ability to induce ER stress [7, 11, 27]. Therefore, we next analyzed sub-cellular localization of PS and PD in glioma GL261 cells. PS and PD differed significantly not only in the rate of internalization but also in subcellular localization. PS co-localized mostly with lysosomes but possibly with other intercellular vesicles as well (Fig. 1a). However, PS was not detected in organelles such as mitochondria, endoplasmic reticulum (ER), Golgi apparatus and nucleus (Fig. 1a). This localization pattern is typical for hydrophilic phthalocyanines due to the lysosome-tropic effect [28] and is in agreement with previous reports, including ours [29, 30].

In contrast, PD accumulated mostly in the ER and Golgi apparatus (Fig. 1b). This is consistent with PD’s amphiphilic property and asymmetrical polarity, and with its previously demonstrated ability to penetrate the lipid bilayer plasma membrane and subsequent redistribution in the organelle membranes [30, 31].

### PS-PDT and PD-PDT induce cell death in cancer cells

**Fig. 2:**
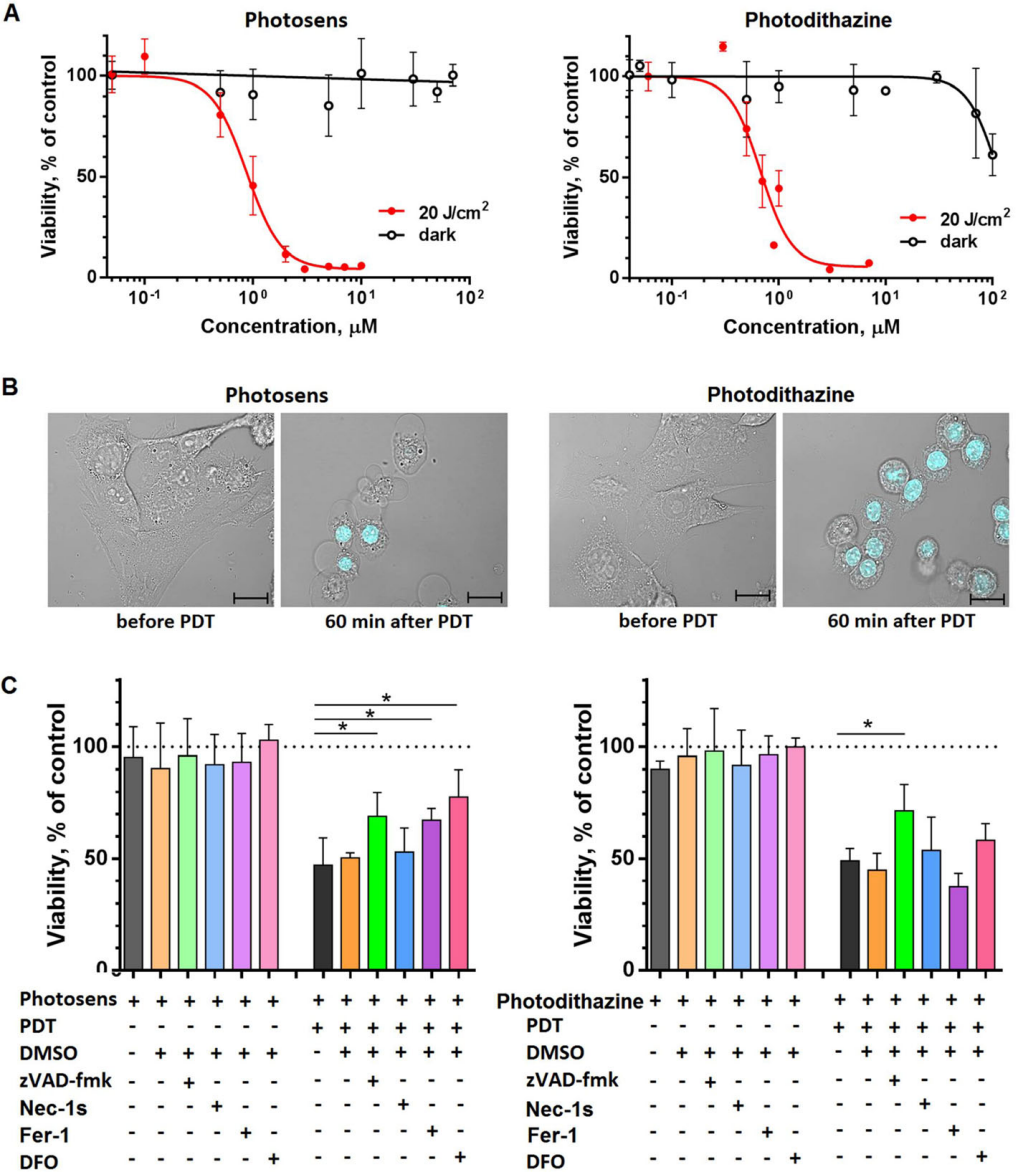
Cell death analysis by MTT assay in cancer cells treated with PDT-PS or PDT-PD. **a** Dark toxicity (black lines) was analyzed after incubating GL261 cells with the respective photosensitizer in serum-free medium for 24 h. For PDT-induced cell death (red lines), cells were first incubated with 10 μM PS or PD in serum-free medium for 4 h and then they were irradiated with a light dose of 20 J/cm^2^ using a LED light source (615–635 nm). MTT assays were performed 24 h after irradiation. ^#^IC_50_ for PS was 0.96 μM [0.79–1.18] and for PD 0.8 μM [0.67–0.92]; the values were calculated with 95% confidence intervals (3 to 5 individual experiments with three replicates in each). **b** Morphology of GL261 cells before and 60 min after PDT. Cells were stained with propidium iodide (blue). Scale bars, 20 μm. **c** Effect of different inhibitors on cell death of GL261 cells induced by PS-PDT or PD-PDT. The following inhibitors were used: 25 μM zVAD-fmk (apoptosis), 20 μM Necrostatin-1 s (necroptosis), and 1 μM Ferrostain-1 or 10 μM DFO (ferroptosis). Cell death in GL261 cells induced by PS-PDT was significantly blocked by zVAD-fmk, ferrostatin-1, and DFO. In contrast, cell death induced by PD-PDT was inhibited only by zVAD-fmk. Cells were first incubated with 10 μM PS or PD in the presence of the respective cell death inhibitor in serum-free medium for 4 h and then the medium was replaced to photosensitizer-free medium followed by irradiation at 20 J/cm^2^ using a LED light source (615–635 nm). After irradiation, the respective inhibitor was added again. MTT assays were performed 13 h after irradiation. Cell viability of the untreated control (no photosensitizer or inhibitor) was set as 100% (dotted line). The values are the means ±SEM. Statistical significance was calculated by using t-criteria with Bonferroni correction, *p < 0.05; ^#^IC_50_ values are given with 95% confidence interval

Next, we analyzed the possibility of inducing cell death in glioma GL261 cells by treatment with PS or PD followed by irradiation with a light dose of 20 J/cm^2^. The control GL261 cells were incubated in the dark with the same doses of photosensitizers for 4 h and then incubated further. Cell death was not induced by PS in concentrations of up to 100 μM in the dark (Fig. 2a) but PD at > 30 μM significantly reduced cell viability (Fig. 2a). Irradiation with a light dose of 20 J/cm^2^ resulted in cell death at concentrations of photosensitizers not exceeding ~ 1 μM (Fig. 2a, b). PS and PD had IC_50_ of 0.96 μM and 0.8 μM, respectively, after irradiation of GL261 cells with a light dose of 20 J/cm^2^.

To determine the type of cell death induced by PS-PDT and by PD-PDT in GL261 cells, use was made of cell death inhibitors that specifically block apoptosis (zVAD-fmk, a pan-caspase blocker), necroptosis (Necrostatin-1 s, a RIPK1 inhibitor) or ferroptosis (Ferrostatin-1, an inhibitor of reactive oxygen species and lipid peroxidation and deferoxamine [DFO], an iron chelator) [32]. It is known that the type of cell death induced by photosensitizers may depend on the photosensitizer, its concentration, and the light dose. At high concentrations or high light doses, photosensitizers may cause an immediate uncontrolled cell death called accidental necrosis. Therefore, we chose treatment conditions corresponding to the IC_50_. After 13 h of PS-PDT, the effect of apoptosis and ferroptosis inhibitors was evident. The pan-caspase inhibitor zVAD-fmk significantly inhibited the death of GL261 cells induced by PS-PDT (Fig. 2c), as well as by Ferrostatin-1 and DFO [33–35], which are specific ferroptosis inhibitors. These data indicate that PS-PDT induces a mixed type of cell death with apoptotic and ferroptotic components. Indeed, it has been reported that PDT can induce mixed forms of cell death [36]. Importantly, the cell death induced by PD-PDT was inhibited only by the apoptosis inhibitor zVAD-fmk but not by Necrostatin-1 s, Ferrostatin-1 or DFO (Fig. 2c), showing that the cells died purely by apoptosis.

### Cell death induced by PS-PDT or PD-PDT is associated with DAMPs emission

**Fig. 3:**
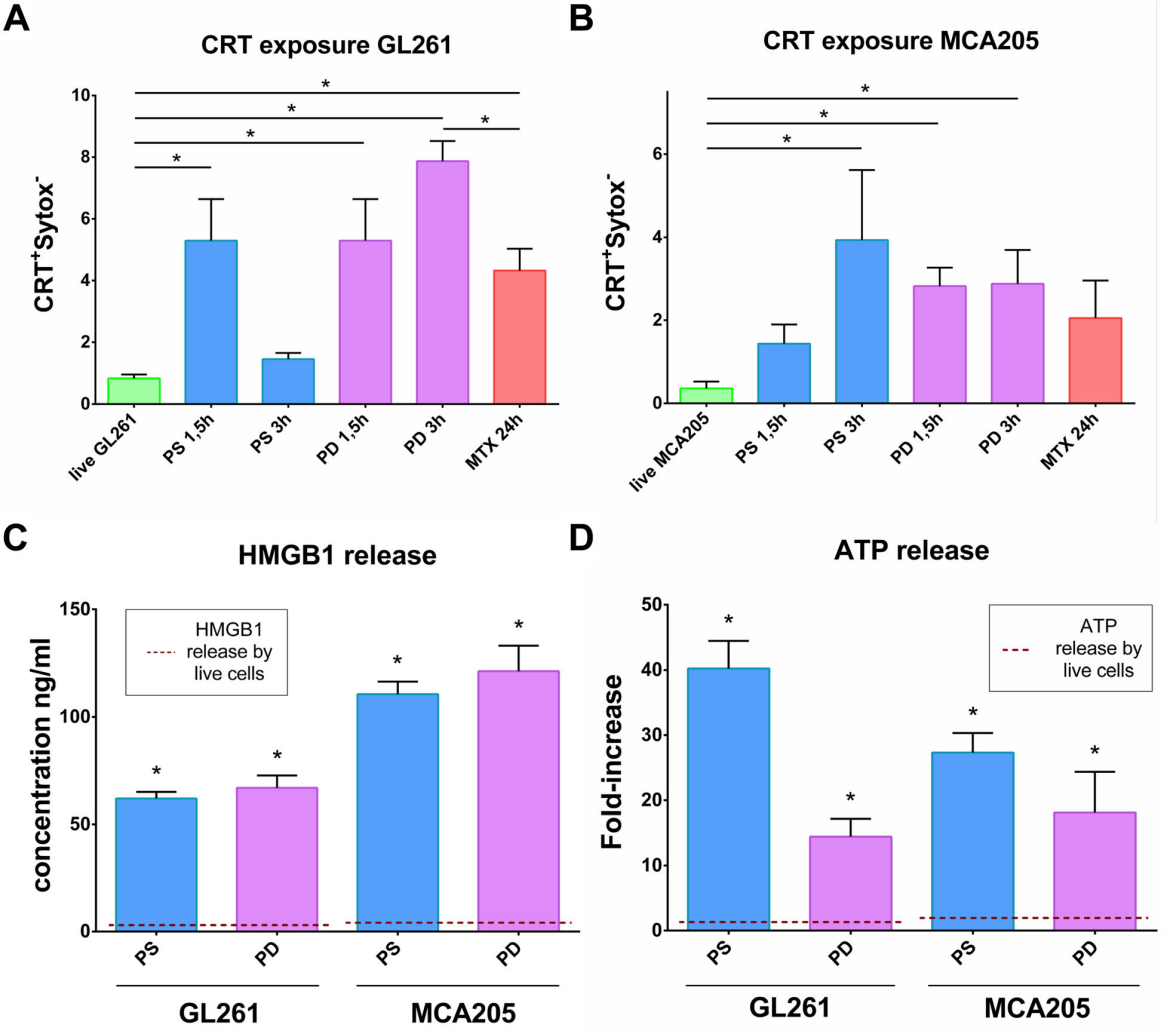
Cell death in cancer cells is associated with CRT exposure at the cell surface and HMGB1 and ATP release. **a** and **b** Quantification of flow cytometry analysis of CRT exposure at the cell surface of Sytox Green negative cells. GL261 (**a**) and MCA205 (**b**) cells were recovered after 1.5 h and 3 h of treatment with either PDT-PS or PDT-PD or left untreated (live). As a positive control, cells were stimulated for 24 h with the ICD inducer, MTX (2 μM). Calreticulin exposure values represent the mean values ± SEM from three independent experiments (each experiment was done in a duplicate). Statistical significance was calculated by using Mann Whitney non-parametric test, *p < 0.05. **c** GL261 and MCA205 cells were recovered for 24 h after PDT-PS or PDT-PD treatment or left untreated (live) and HMGB1 was measured in the supernatants. Cell death was analyzed by an MTT assay, is presented in Additional file 2: Figure S2C. HMGB1 values represent the mean values of four independent experiments. Statistical significance was calculated by Mann Whitney non-parametric test, *p < 0.01. **d** GL261 and MCA205 cells were recovered for 24 h after PDT-PS or PDT-PD treatment or left untreated (live) and ATP was measured in the supernatants. ATP values represent fold increase relative to untreated cells and the mean values of eight independent experiments. Statistical significance was calculated by using Mann Whitney non-parametric test, * *p* < 0.006

One of the main characteristics of ICD is the emission of DAMPs, such as surface exposure of CRT and release of HMGB1 and ATP, which have a beneficial role in anticancer therapy due to their interaction with the innate immune system [4, 37, 38]. In GL261 and MCA205 cells, double staining with Sytox Green, a plasma impermeable dye, and anti-CRT antibodies showed that CRT exposure was a rapid process detectable within 1.5–3 h after PS-PDT or PD-PDT treatment (Fig. 3a, b and Additional file 2: Figure S2A, S2B). Of note, upregulation of CRT at the surface of GL261 cells after PS-PDT or PD-PDT was more pronounced than after MTX, a positive control and a known ICD inducer [3, 39]. We also observed that GL261 and MCA205 cells induced by PS-PDT or PD-PDT release HMGB1 (Fig. 3c) and ATP (Fig. 3d) but this was associated with plasma membrane rupture (Additional file 2: Figure S2C). Thus, both cancer cell lines stimulated with PS-PDT or PD-PDT induce emission of the three crucial DAMPs (CRT, HMGB1 and ATP), which points to the immunogenic nature of cell death.

### Cancer cells killed by PS-PDT or PD-PDT are phagocytosed and induce activation and maturation of BMDCs

**Fig. 4:**
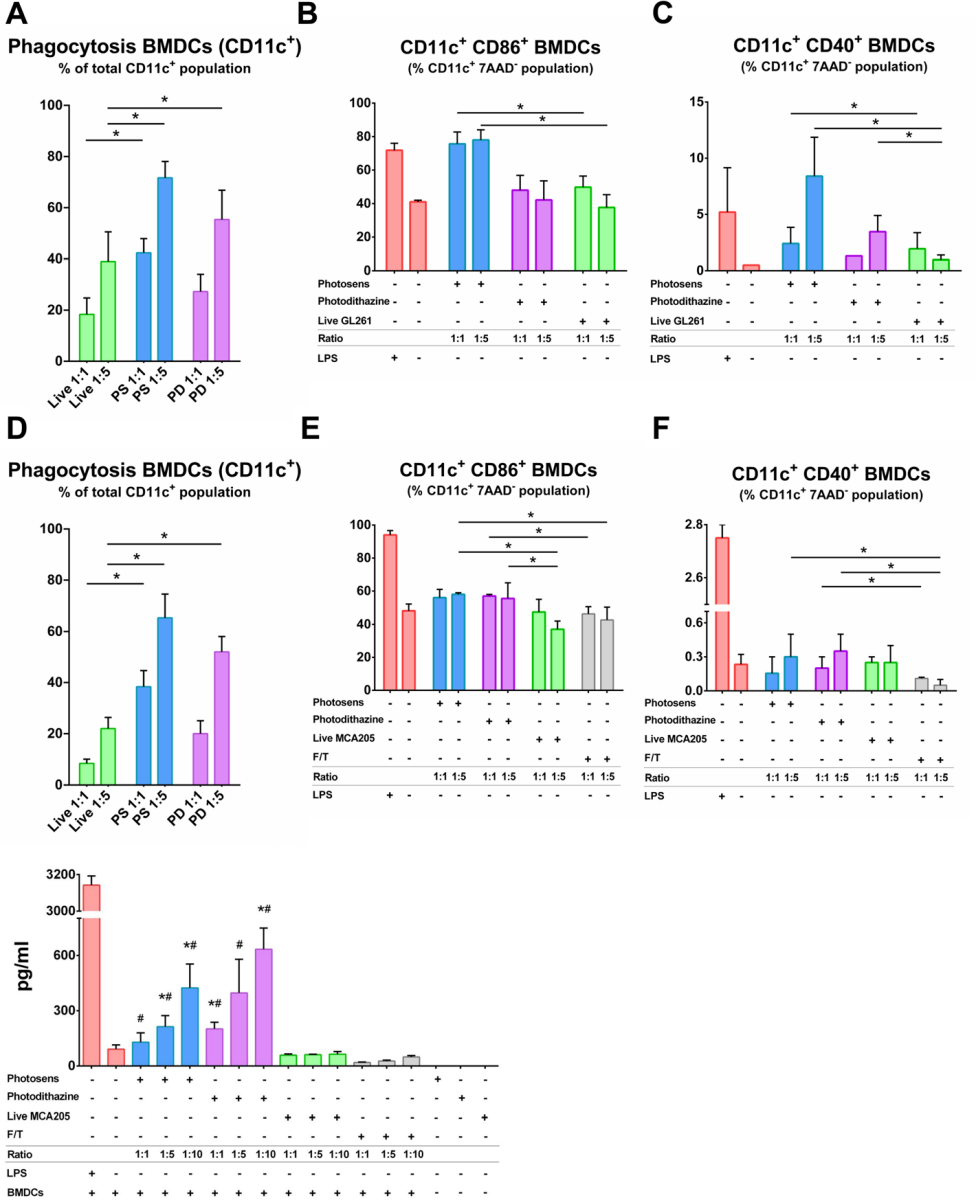
Phagocytosis assay and analysis of BMDCs maturation in vitro. Tumor cells dying after treatment with PS-PDT or PD-PDT were efficiently engulfed by BMDCs in vitro (**a** and **d**). The data for the uptake of GL261 (**a**) and MCA205 (**d**) cells treated with PS-PDT or PD-PDT represent the mean values ± SEM of the duplicates from three independent experiments The rate of phagocytosis increased with the increase in the number of dying/dead cells (1:1 versus 1:5). Statistical significance was calculated by two-way ANOVA, *p < 0.01. Representative flow cytometry dot plots show the uptake of CMFDA-labeled dead GL261 and MCA205 cell material by BMDCs (CD11c^+^CMFDA^+^ double-positive cells) are shown in the Additional file 3: Figure S3A, S3B. **b**-**f** Tumor cells dying after PS-PDT or PD-PDT treatment induce BMDC maturation in vitro. Co-culture of BMDCs with dying GL261 (**b**) and MCA205 (**e**) cells in two different ratios (1:1 and 1:5) and the percentage of CD11c^+^CD86^+^ BMDCs is expressed as the mean value ±SEM. Statistical significance was calculated by a Mann-Whitney non parametric t-test, *p < 0.01. Co-culture of BMDCs with dying GL261 (**c**) and MCA205 (**f**) cells after treatment with PS-PDT or PD-PDT in two different ratios (1:1 and 1:5) and the percentage of CD11c^+^CD40^+^ BMDCs is expressed as the mean value ±SEM of five independent experiments for PS-PDT and four independent experiments for PD-PDT; each experiment was done in the duplicate. In all figure panels, BMDCs stimulated with LPS served as a positive control. MCA205 cells subjected to the several rounds of freeze-thaw (F/T) cycles were used as a negative control in (**e** and **f**). Statistical significance was calculated by a Mann-Whitney non parametric t-test, *p* < 0.05. **g** Absolute concentrations of IL-6 are the mean values ± SEM from three independent experiments in the co-cultures of BMDCs with the respective target MCA205 cells at three different ratios (1:1, 1:5 and 1:10). LPS treated BMDCs were used as a positive control. Statistical significance was calculated by a Mann-Whitney non parametric t-test. The differences are shown by comparing the respective group with BMDCs co-cultured with either *live MCA205 or ^#^F/T MCA205 cells. *p* < 0.03

Phagocytosis of cancer GL261 and MCA205 cells killed by PS-PDT or PD-PDT by BMDCs was analyzed in vitro (Fig. 4a, d and Additional file 3: Figure S3A, S3B)*.* After co-culturing live, untreated cancer cells or PDT-treated cells with BMDCs, only dying cancer cells were effectively engulfed by BMDCs. Increasing the ratio of BMDCs to dead GL261 or MCA205 cells from 1:1 to 1:5 proportionally increased the rate of their engulfment (Fig. 4a, d and Additional file 3: Figure S3A, S3B).

To gain further insight into the functional status of BMDCs, we evaluated the immunogenic properties of GL261 or MCA205 cells killed by PS-PDT or PD-PDT in vitro. Specifically, we compared BMDCs exposed to cancer cells treated with PS-PDT or PD-PDT to BMDCs that were co-cultured with live cells. LPS was used as a positive control. GL261 and MCA205 dying cells treated with PS-PDT induced phenotypic maturation of BMDCs, as indicated by surface upregulation of the co-stimulatory molecule CD86 when compared with BMDCs co-cultured with live cells (Fig. 4b, e). Co-culture with the same amount of PD-PDT-treated dying GL261 cells (Fig. 4c) and MCA205 cells (Fig. 4f) induced CD40 surface expression in a cell ratio-dependent manner only when co-culture was with dying GL261 cells. The substantial surface expression of these molecules was similar to that induced by LPS, a TLR-4 agonist, when co-cultured with dying GL261 cells (Fig. 4b, e). Of note, co-culture with the same amount of MCA205 (Fig. 4e, f) and GL261 (Additional file 4: Figure S4A) cells undergoing accidental necrosis (F/T) did not alter the maturation status of the BMDCs. These findings are in agreement with the previously published data indicating that cancer cells killed by freeze-thaw cycles induce accidental necrosis of cancer cells and is only weakly immunogenic or not immunogenic at all [39–41]. Moreover, dying MCA205 cells after PS-PDT or PD-PDT treatment were able to induce MHC II expression in BMDCs much more efficiently than by F/T or live cells (Additional file 4: Figure S4B).

To get further insight into the functional status of BMDCs, we evaluated the patterns of IL-6 cytokine production. We compared BMDCs exposed to PS-PDT or PD-PDT treated MCA205 cells with those exposed to LPS or respective live cancer MCA205 cells. We found that BMDCs exposed to PS-PDT or PD-PDT treated cancer cells displayed a distinctive and ratio-dependent pattern of functional activation characterized by IL-6^high^ (Fig. 4g). Interestingly, the failure of accidental necrotic cells to stimulate the production of IL-6 by BMDCs points to its non-immunogenic nature (Fig. 4g).

Together, these in vitro results indicate that two different types of cancer cells namely glioma GL261 and fibrosarcoma MCA205 cells treated with PS-PDT or PD-PDT are potent inducers of the phenotypic maturation of BMDCs and their phagocytic capacity.

### Cancer cells treated with PS-PDT or PD-PDT are effective vaccines in vivo

**Fig. 5:**
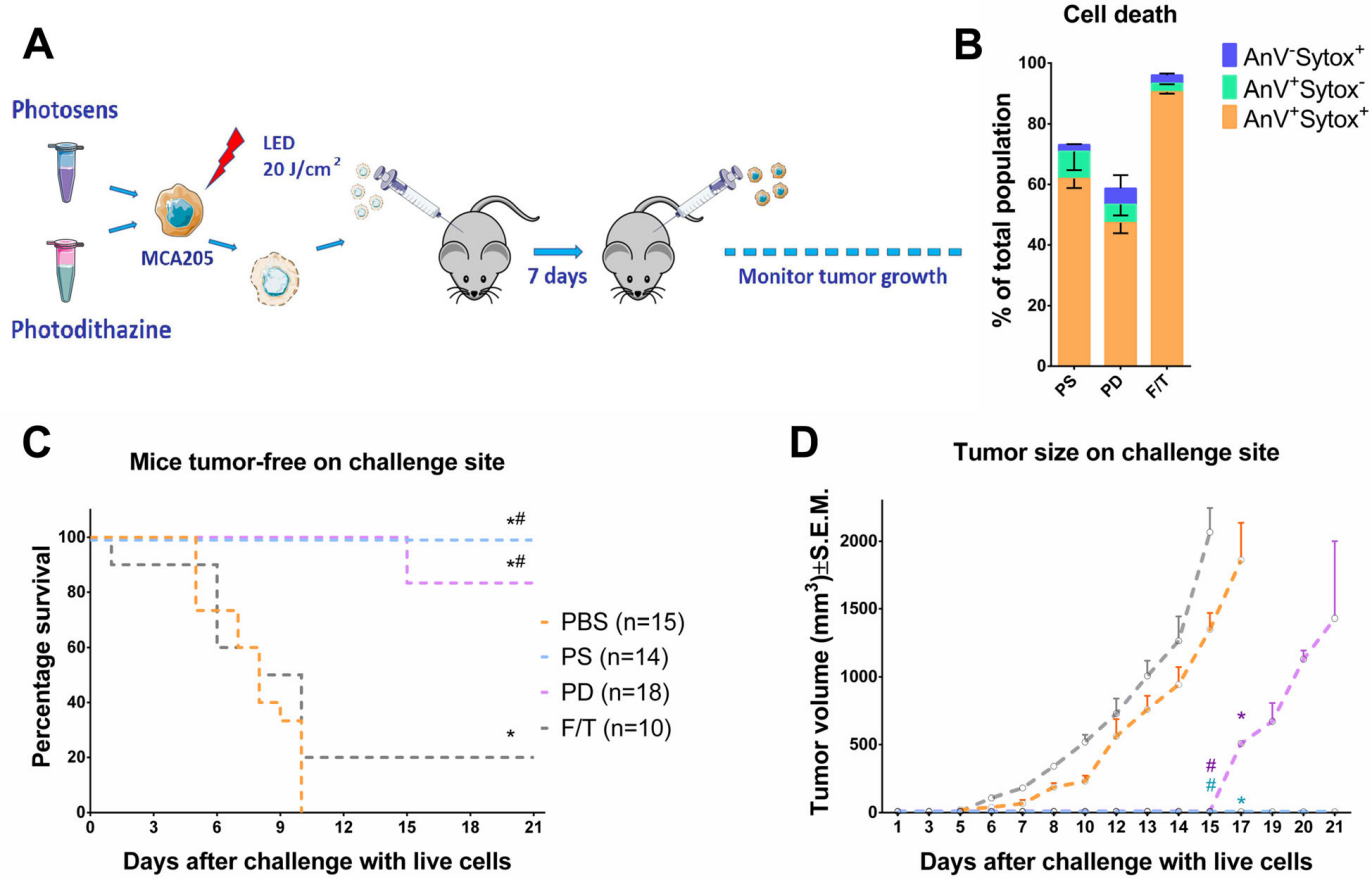
Tumor cells dying after PS-PDT or PD-PDT treatment induce anti-tumor immunogenicity in vivo. **a** In vivo prophylactic tumor vaccination model. **b** Cell death measured by flow cytometry of the cells used for immunization of the mice in (**c**). The cells used for immunization were stimulated with PS-PDT or PD-PDT and re-suspended in PBS before injection. **c** shows the evolution of tumor incidence over time as a Kaplan–Meier curve. MCA205 cells treated with PS-PDT or PD-PDT were used to vaccinate C57BL/6 J mice, which were challenged 1 week later with living cells of the same type. Dying MCA205 cells induced by PS-PDT or PD-PDT triggered an anti-tumor immune response when mice were immunized with 5 × 10^5^ cells. The statistical difference from PBS immunization (negative control) was calculated by a long-rank Manel-Cox test, *p < 0.01. **d** The size of the tumors growing at the challenge site of the mice in the prophylactic tumor vaccination experiments used in (**c**). The statistical differences from PBS immunization or immunization with accidental necrotic cells (F/T) are shown for each vaccination group and was calculated by a Mann-Whitney non parametric t-test, *p < 0.05. *Different from PBS group; ^#^different from F/T group

To investigate the ability of cancer cells treated with PS-PDT or PD-PDT to activate the adaptive immune system, we carried out a well-established in vivo mouse fibrosarcoma MCA205 cancer vaccination experiment in immunocompetent C57BL/6 J mice (Fig. 5a) [42]. The experimental conditions for inducing cell death by PS-PDT and PD-PDT were optimized for the MCA205 cell line, which is conventionally used in this experimental model (data not shown). Next, we immunized C57BL/6 J mice with MCA205 cells that were dying after PS-PDT or PD-PDT treatment (Fig. 5b). Negative control mice were injected with PBS [42] or with MCA205 cells undergoing accidental necrosis. The immunized mice were then challenged with live MCA205 tumor cells. Protection against tumor growth at the challenge site was interpreted as a sign of successful priming of the adaptive immune system. Mice immunized with MCA205 cells treated with PS-PDT or PD-PDT showed signs of robust activation of the adaptive immune system and protection against tumor growth. By contrast, there was tumor growth in most of the mice immunized with PBS (Fig. 5c), which confirms our in vitro findings and points to the strong immunogenic properties of cancer cells treated with PS-PDT or PD-PDT. Moreover, the tumors growing at the challenge site of the PBS-vaccinated mice was large in size and occurred earlier (Fig. 5d), confirming that dying cancer cells are strongly immunogenic in vivo. Notably, the mice that were vaccinated with the same number of F/T cells developed more tumors at the challenge site (Fig. 5c and d), confirming the previously published findings that accidental necrotic cells are less immunogenic [39]. These data indicate that induction of death in cancer cells by PS-PDT or PD-PDT activates an adaptive immune response, which is one of the important properties of ICD.

## Discussion

Our results demonstrate that cancerous cells undergoing cell death after PS-PDT or PD-PDT can be immunogenic. This immunogenicity was shown in vitro by co-culturing glioma GL261 or fibrosarcoma MCA205 cells with BMDCs, whereupon the dead cancer cells were efficiently phagocytized and resulted in phenotypic maturation of BMDCs, as well as in vivo, where MCA205 cells served as a potent vaccine in a prophylactic tumor vaccination model. Therefore, cancer cells killed by PS-PDT or PD-PDT appear to be potent inducers of an adaptive immune response and mediators of effective anti-tumor immunity.

PDT is a unique anti-cancer therapy involving a photosensitizing agent, photoexciting light and molecular oxygen. It is characterized by local generation of singlet oxygen and other cytotoxic oxidants generating oxidative stress with subsequent cell death [16]. We found that the apoptosis inhibitor zVAD-fmk as well as several ferroptosis inhibitors (Ferrostatin-1 and DFO) significantly inhibited the cell death induced by PS-PDT but the necroptosis inhibitor necrostatin-1 s did not. However, the cell death induced by PD-PDT was blocked only by the apoptosis inhibitor (zVAD-fmk). These data raise the possibility that PS-PDT induces features of apoptotic and ferroptotic cell death in GL261 cells. Ferroptosis is a regulated type of cell death resulting from iron accumulation and lipid peroxidation, which can be blocked by Ferrostatin-1 and DFO [33, 35, 43]. Ferrostatin-1 is an aromatic amine that specifically binds to lipid reactive oxygen species (ROS) and protects cells from lipid peroxidation, whereas DFO has a high affinity for extracellular free iron, which is directly involved in ROS production. Our results suggest a relationship between PS-PDT-induced death and ferroptosis, and especially the involvement of lipid ROS. Of interest, we found that PS specifically accumulated in lysosomes in GL261 cells. It has recently been shown that lysosomal activity is tightly linked with ferroptosis by modulation of the iron equilibrium and ROS metabolism [44, 45]. These data are in line with previous reports pointing to PDT as an inducer of several types of cell death within the same cell population [36, 46].

It has been shown that the ability to induce ICD is associated with localization of the photosensitizers or drugs in the ER and their ability to induce ER stress [7, 11, 27]. In PDT, hypericin is a photosensitizer that localizes predominantly in the ER and Golgi apparatus [47] and it induces ICD, which is dependent on the induction of ER stress [7, 11, 27]. Indeed, we found that PD accumulated mainly in the ER and Golgi apparatus, suggesting the involvement of ER in the immunogenicity induced by PD-PDT. However, in contrast to PD, PS was localized mainly in lysosomes. Therefore, mechanisms other than those associated with ER might play a role in PS-induced immunogenicity. Of note, ER-independent immunogenicity has also been described [39]. These findings confirm the notion that subcellular localization of a photosensitizer is highly dependent on its nature [46].

The findings presented here confirm that PDT destroys tumors not only by directly killing tumor cells, but also involves an important immunological component, including the induction of ICD. On the one hand, we have demonstrated the immunogenicity of cancerous cells (i.e. glioma GL261 and fibrosarcoma MCA205) killed by PS-PDT or PD-PDT in vitro. These cancer cells induced by PDT using the novel photosensitizers (i.e., PS and PD) induced emission of the crucial DAMPs such as CRT, HMGB1 and ATP. These dying cancer cells were efficiently engulfed by BMDCs, leading to their phenotypic activation in vitro and production of IL-6 in a cell ratio-dependent manner. Of interest, that it has been previously found that IL-6 is necessary for calreticulin mediated priming of Th17 cells and inhibiting the generation of Treg cells [48] and Th17 cells play an important role in establishing of anti-tumor immunity [49].

These results add PS and PD to a list of photosensitizers capable of inducing ICD [46], which could be relevant in the treatment of brain tumors, including gliomas. Moreover, we used fibrosarcoma MCA205 cells, which have been extensively used to characterize ICD [50–52], to confirm that dying cancer cells induced by PS-PDT or PD-PDT are immunogenic in the co-culture assays with BMDCs in vitro and in the mouse tumor prophylactic vaccination model. It is important to mention that in order to better recapitulate the cancer patient’s situation and to establish a therapeutic effect, dying cancer cells needed to be injected into mice with already existing tumors. Indeed, in the recently published study, it has been shown that injection of dying necroptotic cancer cells directly into the tumor bed led to a more effective control of tumor growth in mice [53]. Therefore, in future work it would be interesting to analyze whether cancer cells treated with PS-PDT or PD-PDT can be used as a vaccine in mice with established tumors. To date, the observation of immunogenic dying cancer cells is restricted to immunogenic apoptosis [1, 5, 50, 54, 55] and necroptosis [39, 56–58]. However, based on this study, we suggest that cancerous cells dying with at least some features of ferroptosis can be also immunogenic [59]. Future studies are needed to provide detailed insights into the immunogenic properties of ferroptotic cancerous cells.

## Conclusions

These results identify PS and PD as novel immunogenic cell death inducers in vitro and in vivo that could be effectively combined with PDT in cancer therapy.

## Supplementary information

**Additional file 1: Figure S1.** Chemical structure, absorption, fluorescence spectra and dynamics of PS and PD uptake by GL261 cells. (A) Photosens (a mixture of di-, tri- and tetrasubstituted fractions of aluminum phthalocyanine, the number of sulfonic groups is 3.4). (B) Photodithazine (bis-N-methylglucamine salt of chlorin *e6*). Absorbance and fluorescence spectra obtained by spectrofluorometry are on the right. (C) Cellular uptake of PS and PD was assessed by confocal microscopy during up to 4 h of incubation with 10 μM PS or PD. Fluorescence images were obtained at λ_ex_ 633 nm and λ_em_ 650–710 nm; scale bars 20 μm. (D) The fluorescence signal in cells incubated with PS or PD expressed as mean ± SD (*n* ≥ 10). The fluorescence signal intensity (I_fl_) before the photosensitizers were added did not exceed 0.3 a.u.

**Additional file 2: Figure S2.** Analysis of expression of CRT in GL261 and MCA205 cells treated with PS and PD by flow cytometry. Representative dot plots of GL261 (A) and MCA205 (B) cells treated with different photosensitizers are shown. The results were compared to mitoxantrone – treated cells (a positive control) and viable cells. CRT V450 positive cells (blue) were compared to isotype control stained cells (grey) and gated to analyze the expression of CRT on the surface of cells showing an intact membrane permeability when stained with Sytox Green. The following concentrations of photosensitizers were used for glioma GL261: 1.4 μM PS or 1.2 μM PD and for fibrosarcoma MCA205: 1.5 μM PS or 1.8 μM PD.

**Additional file 3: Figure S3.** Phagocytosis assay and cell death analysis. (A, B) Flow cytometry analysis of phagocytosis of dying GL261 and MCA205 cells after PDT-PS or PDT-PD treatment by BMDCs. Representative flow cytometry dot plots show the uptake of CMFDA-labeled dead GL261 (A) and MCA205 (B) cell material by BMDCs (CD11c^+^CMFDA^+^ double-positive cells). (C) Analysis of cell death in GL261 and MCA205 cells. Cell death was measured by an MTT assay.

**Additional file 4: Figure S4.** Analysis of BMDCs maturation in vitro*.* (A) Co-culture of BMDCs with accidental necrotic F/T MCA205 cells in two different ratios (1:1 and 1:5). LPS-treated BMDCs were used as a positive control. Percentage of CD11c^+^CD86^+^ BMDCs expressed as the mean value + SEM of four independent experiments. (B) Co-culture of BMDCs with dying MCA205 cells treated with PS-PDT or PD-PDT in three different ratios (1:1, 1:5, 1:10). LPS-treated BMDCs were used as a positive control. Percentage of CD11c^+^MHC II^+^ BMDCs expressed as the mean value ±SEM of three independent experiments performed in duplicates. Statistical significance was calculated by a Mann-Whitney non parametric t-test, * *p* < 0.05.

## Data Availability

All raw data are available in the Institute of Biology and Biomedicine at National Research Lobachevsky State University of Nizhni Novgorod and in the Cell Death Investigation and Therapy Laboratory at Ghent University.

## Abbreviations

ATP: Adenosine triphosphate
BMDCs: Bone-marrow derived dendritic cells
CRT: Calreticulin
DAMPs: Damage-associated molecular patterns
ER: Endoplasmic reticulum
F/T: Freeze and thaw
GM-CSF: Granulocyte-macrophage colony-stimulating factor
HMGB1: High mobility group box 1
ICD: Immunogenic form of cancer cell death
LPS: Lipopolysaccharide
MHCII: Major histocompatibility complex class II
PBS: Phosphate buffered saline
PD: Photodithazine
PDT: Photodynamic therapy
PS: Photosens
ROS: Reactive oxygen species
