# Supplementary figures and images for: Immunogenic cell death induced by a new photodynamic therapy based on photosens and photodithazine
Source: J Immunother Cancer. 2019 Dec 16;7:350. doi: 10.1186/s40425-019-0826-3 (PMC6916435; doi:10.1186/s40425-019-0826-3)

## Slide 1
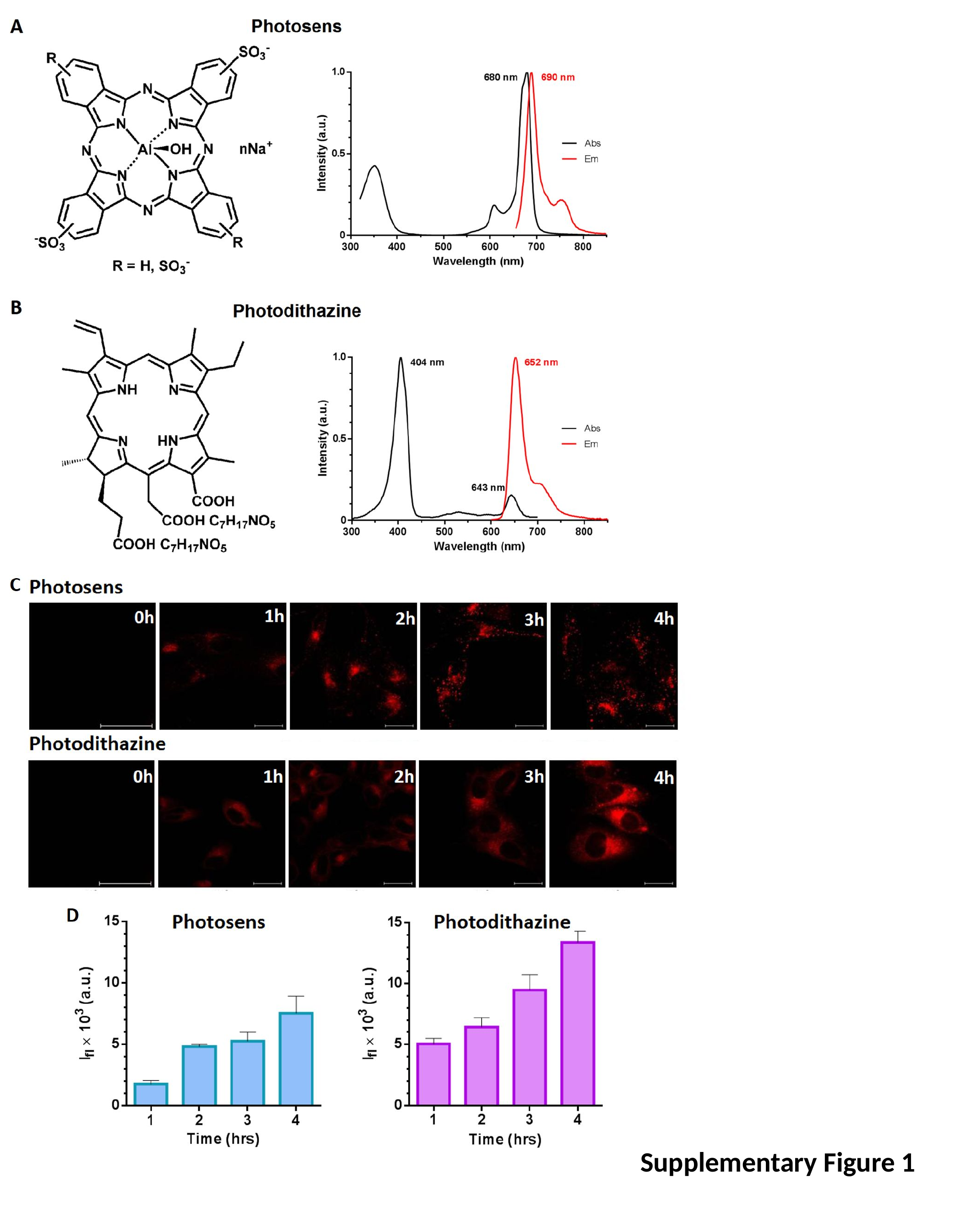

Supplementary Figure 1

Supplement: Supplementary file 1 — Additional file 1: Figure S1. Chemical structure, absorption, fluorescence spectra and dynamics of PS and PD uptake by GL261 cells. (A) Photosens (a mixture of di-, tri- and tetrasubstituted fractions of aluminum phthalocyanine, the number of sulfonic groups is 3.4). (B) Photodithazine (bis-N-methylglucamine salt of chlorin e6). Absorbance and fluorescence spectra obtained by spectrofluorometry are on the right. (C) Cellular uptake of PS and PD was assessed by confocal microscopy during up to 4 h of incubation with 10 μM PS or PD. Fluorescence images were obtained at λex 633 nm and λem 650–710 nm; scale bars 20 μm. (D) The fluorescence signal in cells incubated with PS or PD expressed as mean ± SD (n ≥ 10). The fluorescence signal intensity (Ifl) before the photosensitizers were added did not exceed 0.3 a.u. [file 40425_2019_826_MOESM1_ESM.pptx]

## Slide 1
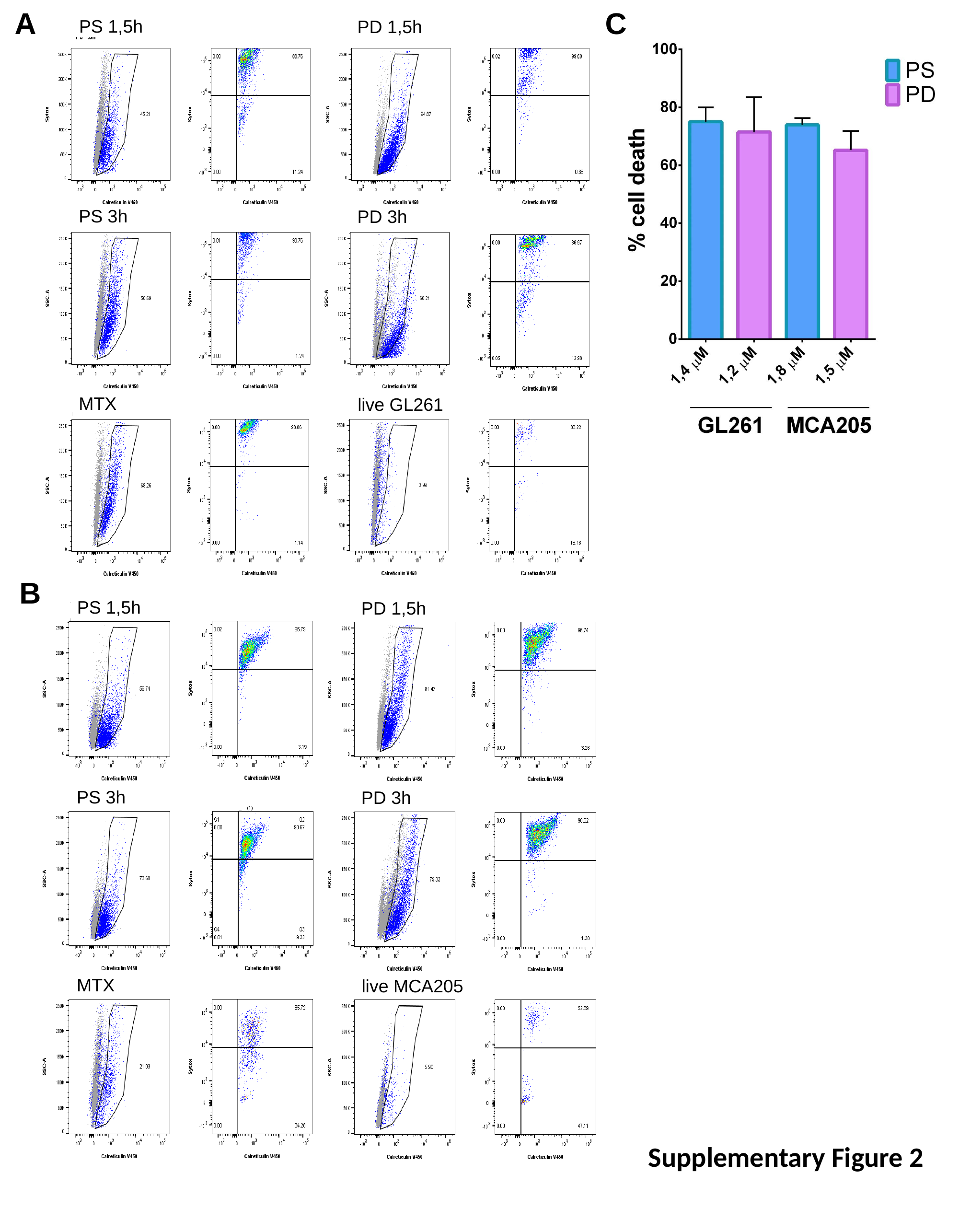

A
B
Supplementary Figure 2
C
PS 1,5h
PD 1,5h
PS 3h
PD 3h
MTX
live GL261
PS 1,5h
PD 1,5h
PS 3h
PD 3h
MTX
live MCA205

Supplement: Supplementary file 2 — Additional file 2: Figure S2. Analysis of expression of CRT in GL261 and MCA205 cells treated with PS and PD by flow cytometry. Representative dot plots of GL261 (A) and MCA205 (B) cells treated with different photosensitizers are shown. The results were compared to mitoxantrone – treated cells (a positive control) and viable cells. CRT V450 positive cells (blue) were compared to isotype control stained cells (grey) and gated to analyze the expression of CRT on the surface of cells showing an intact membrane permeability when stained with Sytox Green. The following concentrations of photosensitizers were used for glioma GL261: 1.4 μM PS or 1.2 μM PD and for fibrosarcoma MCA205: 1.5 μM PS or 1.8 μM PD. [file 40425_2019_826_MOESM2_ESM.pptx]

## Slide 1
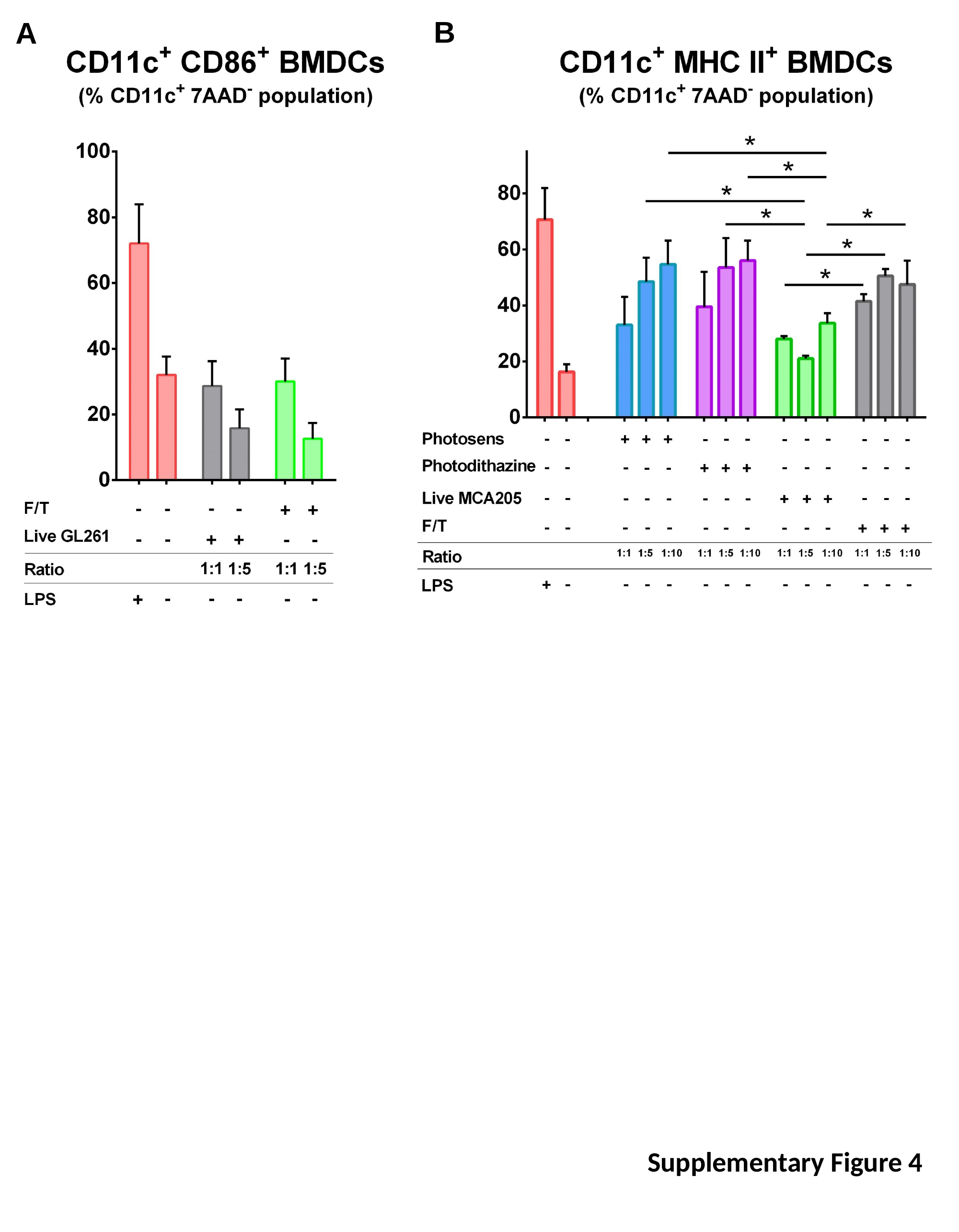

B
A
Supplementary Figure 4

Supplement: Supplementary file 4 — Additional file 4: Figure S4. Analysis of BMDCs maturation in vitro. (A) Co-culture of BMDCs with accidental necrotic F/T MCA205 cells in two different ratios (1:1 and 1:5). LPS-treated BMDCs were used as a positive control. Percentage of CD11c+CD86+ BMDCs expressed as the mean value + SEM of four independent experiments. (B) Co-culture of BMDCs with dying MCA205 cells treated with PS-PDT or PD-PDT in three different ratios (1:1, 1:5, 1:10). LPS-treated BMDCs were used as a positive control. Percentage of CD11c+MHC II+ BMDCs expressed as the mean value ±SEM of three independent experiments performed in duplicates. Statistical significance was calculated by a Mann-Whitney non parametric t-test, * p < 0.05. [file 40425_2019_826_MOESM4_ESM.pptx]
