# Supplementary material for: Immunogenic cell death induced by a new photodynamic therapy based on photosens and photodithazine
Source: J Immunother Cancer. 2019 Dec 16;7:350. doi: 10.1186/s40425-019-0826-3 (PMC6916435; doi:10.1186/s40425-019-0826-3)
Supplement: Supplementary file 3 — Additional file 3: Figure S3. Phagocytosis assay and cell death analysis. (A, B) Flow cytometry analysis of phagocytosis of dying GL261 and MCA205 cells after PDT-PS or PDT-PD treatment by BMDCs. Representative flow cytometry dot plots show the uptake of CMFDA-labeled dead GL261 (A) and MCA205 (B) cell material by BMDCs (CD11c+CMFDA+ double-positive cells). (C) Analysis of cell death in GL261 and MCA205 cells. Cell death was measured by an MTT assay. [file 40425_2019_826_MOESM3_ESM.pptx]

## Slide 1
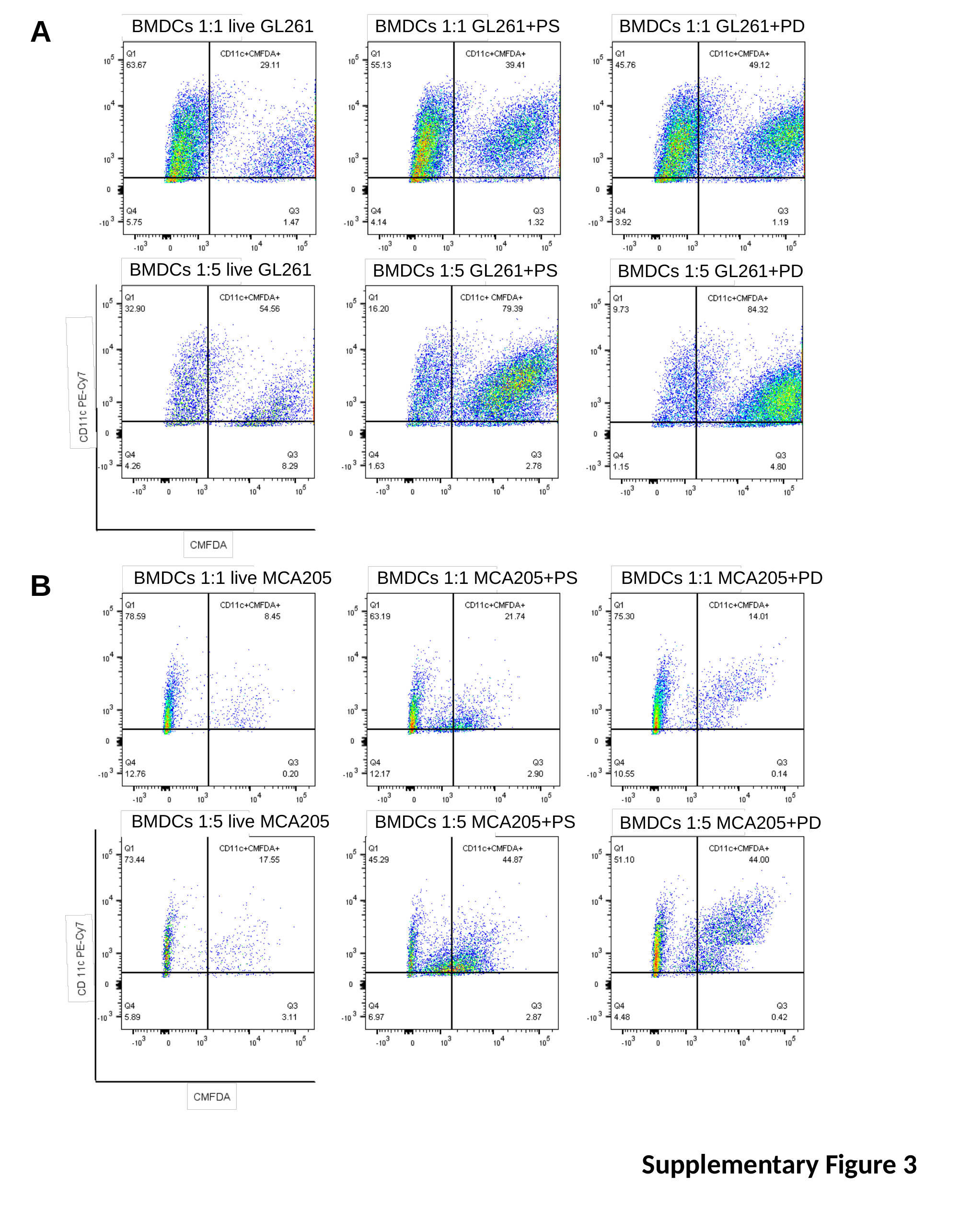

A
B
Supplementary Figure 3
BMDCs 1:1 live GL261
BMDCs 1:1 GL261+PS
BMDCs 1:1 GL261+PD
BMDCs 1:5 live GL261
BMDCs 1:5 GL261+PS
BMDCs 1:5 GL261+PD
BMDCs 1:1 live MCA205
BMDCs 1:1 MCA205+PS
BMDCs 1:1 MCA205+PD
BMDCs 1:5 live MCA205
BMDCs 1:5 MCA205+PS
BMDCs 1:5 MCA205+PD
